# Commentary: Synaptic vesicle glycoprotein 2C (SV2C) modulates dopamine release and is disrupted in Parkinson disease

**DOI:** 10.3389/fnsyn.2017.00018

**Published:** 2018-01-04

**Authors:** Sachchida Nand Rai, Hareram Birla, Walia Zahra, Saumitra Sen Singh, Surya Pratap Singh

**Affiliations:** Research Scholar, Department of Biochemistry, Institute of Science, Banaras Hindu University, Varanasi, India

**Keywords:** Parkinson disease, dopamine, synapses, SV2C, basal ganglia

It is well-known that high levels of Synaptic vesicle 2C proteins (SV2C) were found primarily in old brain regions like pallidum, substantia nigra, midbrain, brainstem, and olfactory bulb. They have also show that SV2C level was relatively low in the cerebellar cortex and untraceable in the hippocampus and cerebral cortex. Synaptic vesicle protein family members are closely related and they are distributed in general (SV2A) or in a very restricted manner (SV2C) depending on their functional regulation of synaptic vesicle proteins (Janz and Südhof, 1999).

In all neurons, synaptic vesicle 2 proteins, SV2A, SV2B, and SV2C are localized as integral protein on the surface of synaptic vesicles. In basal ganglia, particularly within the dopaminergic neurons in substantia nigra pars compacta and the ventral tegmental area, SV2C was densely expressed (more than 70%). Dardou et al. have suggested that SV2C may contribute significantly in the regulation of neurotransmitter release and synaptic transmission in the basal ganglia, including cholinergic striatal interneurons and nigrostriatal/mesolimbic dopamine neurons (Dardou et al., 2011).

Several lines of evidence have suggested that nicotine (environmental modulators) mediated protection of Parkinson's disease (PD) is due to genetic modulation of the SV2c gene (Hernán et al., 2002; Hill-Burns et al., 2013). Another group also predicted the PD patients' sensitivity to L-DOPA due to variation within the SV2c gene (Altmann et al., 2016). These findings proposed that SV2C may play critical functions in the basal ganglia, although further research work is needed to establish the hypothesis like SV2C mediated chemical effects of nicotine, or to PD.

In this paper, Dunn et al. have established probable alterations in SV2C following dopaminergic cell loss via characterization of SV2C expression in multiple mouse models of PD. This group developed SV2C knock-out (SV2C-KO) mice and proved that SV2C plays a significant role in dopamine function inside the basal ganglia. They have also performed HPLC quantification of dopamine content and its metabolites in SV2C-KO mice model. Authors also investigated the alternation in motor behavior and dopamine content in PD due to loss of α-Synuclein (α-Syn) and subsequent interaction with SV2C. In the presence of nicotine, SV2C-KO mice show a significant decrease in dopamine release in the dorsal striatum compared to wild-type mice. Lastly, they link the altered expression of SV2C with a mutation in α-Syn in the basal ganglia of postmortem neurodegenerative cases including PD (Dunn et al., 2017).

Dunn et al. proposed that alteration in SV2C significantly affect the dopamine release and neurotransmission leads to PD pathogenesis. SV2C is expressed in the vesicles of dopaminergic neurons and there is a significant reduction of synaptic release of dopamine following genetic deletion of SV2C, this cause reduced motor activity in PD. They have suggested the isolated neurochemical response to nicotine in SV2C-KO mice and the neuroprotective effect of nicotine is mediated through SV2C. Finally, they have demonstrated that in humans with PD and in mice that express mutated α-Syn, there is a specific and significant disruption of SV2C expression. Collectively, this paper has suggested that SV2C plays a significant role as a mediator of dopamine homeostasis and acts as a possible and potent player in the pathogenesis of PD (Dunn et al., 2017).

PD is characterized by death of neurons in the mesencephalon that utilize dopamine as a neurotransmitter for synaptic communication. If dopamine is not properly stored and metabolized it causes severe cytotoxicity to dopaminergic neurons. Thus, the researchers have tried to target any of the pathways that tightly control this neurotransmitter and holds greater therapeutic expectations (Bisaglia et al., 2013). In this regard, SV2C proved to be a potential therapeutic candidate in dopamine homeostasis and search for another molecule which has the same activity in PD is needed.

The paper published by Dunn et al. does explain the potential role of SV2C in the pathogenesis of human neurodegenerative diseases. Although this article does not show any potential relationship between VMAT2 and SV2C since both are involved in dopamine homeostasis. Authors have gone through the idea of making SV2C-KO mice, and then they should also have thought of making VMAT2-KO mice too. This is suggested because both VMAT2 and SV2C are equally responsible for taking up the free dopamine from the cytosol, store it into synaptic vesicles and release at the time of requirement, hence playing a synergistic role in maintaining dopamine homeostasis. Moreover, authors have also not observed the relationship between SV2C and MAO-B. MAO-B converts free dopamine present in the cytosol to toxic dopamine metabolite, thereby causing trouble in L-Dopa therapy given to PD patients. Additionally, the level of SV2C, VMAT2, and MAO-B should have been studied first at the transcriptional level in healthy and control mice, and then SV2C-KO mice should have been made to justify their results more appropriately. This would have resulted in a correct understanding of dopamine homeostasis. Authors should also have seen the effect of the α-Syn mutation on VMAT 2 expression too, as the mutation might result in decreased VMAT2 expression along with SV2C expression.

Overall, this interesting study opens a window to search for therapeutic modifiers which can modulate expression of SV2C in dopaminergic neurons to inhibit PD pathogenesis which is subject to further investigations.

## Author contributions

All authors listed have made a substantial, direct and intellectual contribution to the work, and approved it for publication.

### Conflict of interest statement

The authors declare that the research was conducted in the absence of any commercial or financial relationships that could be construed as a potential conflict of interest.
